# The question of screening organ donors for hepatitis e virus: a case report of transmission by kidney transplantation in France and a review of the literature

**DOI:** 10.1186/s12985-024-02401-2

**Published:** 2024-06-12

**Authors:** Justine Solignac, Celine Boschi, Vincent Pernin, Virginie Fouilloux, Anne Motte, Sarah Aherfi, Maxime Fabre-Aubrespy, Tristan Legris, Philippe Brunet, Philippe Colson, Valérie Moal

**Affiliations:** 1 Centre de Néphrologie Et Transplantation Rénale, Aix Marseille Université, Publique Hôpitaux de Marseille, Hôpital Conception, 147 Boulevard Baille, 13005 Marseille, France; 2 IHU Méditerranée Infection, Publique Hôpitaux de Marseille, 19-21 Boulevard Jean Moulin, 13005 Marseille, France; 3 Aix Marseille Université, Institut de Recherche Et Développement, Microbes Evolution Phylogeny and Infections, 27 Boulevard Jean Moulin, 13005 Marseille, France; 4 Department of Nephrology Dialysis and Kidney Transplantation, Lapeyronie University Hospital, Montpellier, France; 5 Institute for Regenerative Medicine and Biotherapy (IRMB), Montpellier, France; 6 Department of Congenital and Pediatric Cardiac Surgery, Timone Children’s Hospital, Marseille, France; 7 Department of Orthopaedic Surgery, Sainte-Marguerite University Hospital, Marseille, France; 8 Centre de Néphrologie Et Transplantation Rénale, Publique Hôpitaux de Marseille, Hôpital Conception, Marseille, France

**Keywords:** Hepatitis E, Hepatitis E Virus, Organ transplantation, Chronic hepatitis, Donor screening, Donor-derived infection, Transplant-related infection, Donor-derived transmission

## Abstract

**Background:**

Hepatitis E is a potentially serious infection in organ recipients, with an estimated two-thirds of cases becoming chronic, and with a subsequent risk of cirrhosis and death. In Europe, transmission occurs most often through the consumption of raw or undercooked pork, more rarely through blood transfusion, but also after solid organ transplantation. Here we describe a case of Hepatitis E virus (HEV) infection transmitted following kidney transplantation and review the literature describing cases of HEV infection transmitted by solid organ transplantation.

**Case presentation:**

Three weeks after kidney transplantation, the patient presented with an isolated minimal increase in GGT and hepatic cytolysis 6 months later, leading to the diagnosis of genotype 3c hepatitis E, with a plasma viral load of 6.5 log_10_IU/mL. In retrospect, HEV RNA was detected in the patient's serum from the onset of hepatitis, and in the donor's serum on the day of donation, with 100% identity between the viral sequences, confirming donor-derived HEV infection. Hepatitis E had a chronic course, was treated by ribavirin, and relapsed 10 months after the end of treatment.

**Discussion:**

Seven cases of transmission of HEV by solid organ transplantation have been described since 2012 without systematic screening for donors, all diagnosed at the chronic infection stage; two patients died. HEV organ donor transmission may be underestimated and there is insufficient focus on immunocompromised patients in whom mild liver function test impairment is potentially related to hepatitis E. However, since HEV infection is potentially severe in these patients, and as evidence accumulates, we believe that systematic screening of organ donors should be implemented for deceased and living donors regardless of liver function abnormalities, as is already the case in the UK and Spain. In January 2024, the French regulatory agency of transplantation has implemented mandatory screening of organ donors for HEV RNA.

**Supplementary Information:**

The online version contains supplementary material available at 10.1186/s12985-024-02401-2.

## Introduction

Awareness of hepatitis E virus (HEV) has increased considerably over the past 15 years. Autochthonous hepatitis E in developed countries has been increasingly recognized as a significant public health concern [1, 2]. Seroprevalence studies in Europe have shown substantial differences between geographical areas, which can at least in part rely on tested populations and serological tests. In France, for example, seroprevalence has been reported to range from 8.0% to 86.4% in the general population, and from 21.9% to 71.3% in blood donors with a higher endemicity level in southern France [3, 4].

HEV is a small quasi-enveloped (since not enveloped when excreted in stools) RNA virus [5, 6]. Eight genotypes have been identified in the *Paslahepevirus balayani* species in the family *Hepeviridae* and subfamily *Orthohepevirinae*, of which types HEV-1, HEV-2, HEV-3, HEV-4, and HEV-7 are known to infect humans [7]. Recent studies have reported that another species, *Rocahepevirus ratti*, may also infect humans [8, 9].

HEV-1 and HEV-2 have a strictly human reservoir, while that for HEV-3, HEV-4 and HEV-7 is animal [5]. In Europe, pigs and wild boars are the main reservoirs for HEV-3 and HEV-4. Camels are a reservoir for HEV-7 [10]. Very recently, the animal reservoir of human HEV infections has been supplemented by rats, for *Rocahepevirus ratti* [11].

HEV-1 and HEV-2 are responsible for epidemics in developing countries through fecal–oral transmission. In contrast, zoonotic transmission through close contact or – primarily –through consumption of raw or insufficiently cooked food is responsible for HEV infections, especially in developed countries for HEV-3 and HEV-4 genotypes (anecdotally for the HEV-7 genotype in the United Arab Emirates [10]).

Hepatitis E is a potentially serious infectious disease in specific populations. Most often asymptomatic, HEV infection can lead to acute hepatitis, more rarely to death, while HEV-3, HEV-4, HEV-7 and *Rocahepevirus ratti* can lead to chronic infection (defined as viral RNA positivity in the blood and disturbance of liver function tests for more than 3–6 months) in immunocompromised individuals [12–15]. Since 2008, HEV-3 and HEV-4 infections have been recognized as a cause of progression to cirrhosis in these patients, especially in solid organ transplantation recipients (SOTRs) [16–19]. More recently, in 2016, HEV-7 was associated with a chronic infection in a liver transplant recipient from the United Arab Emirates [10]. A first chronic infection by *Rocahepevirus ratti* has been described in 2018 in a liver transplant recipient who lived in a rat-infested housing estate in Hong Kong [20]. Although different studies show a variation in the rate of developing chronicity, overall, HEV infections of *Paslahepevirus balayani* species become chronic in about two-thirds of SOTRs based on large cohort studies and meta-analyses, including in our geographical area [21–24]. Regarding the *Rocahepevirus ratti* species, only one small series of immunocompromised patients has been reported, with a seemingly similar chronicity rate (7/9 patients developed a chronic form) [9].

In addition to the modes of transmission described above, HEV transmission can be iatrogenic. Several studies of blood banks in Europe have shown a prevalence of HEV RNA positivity ranging from 0.02 to 0.14% in blood donors [13], and transfusion-transmitted hepatitis E has been described [25]. Because HEV viremic donors can be asymptomatic, HEV RNA screening is the only option to control transmission (anti-HEV antibodies can be absent in the presence of HEV RNA in cases of very recent infection), and several European countries, including France, have implemented mandatory screening of blood donors for HEV RNA [26]. Moreover, solid organ donation has also been shown to be a possible source of HEV [27–29]. Here, we describe a case of hepatitis E transmission from a kidney transplant donor in France and we discuss the relevance of systematic screening prior to organ donation.

## Case report

**Fig. 1:**
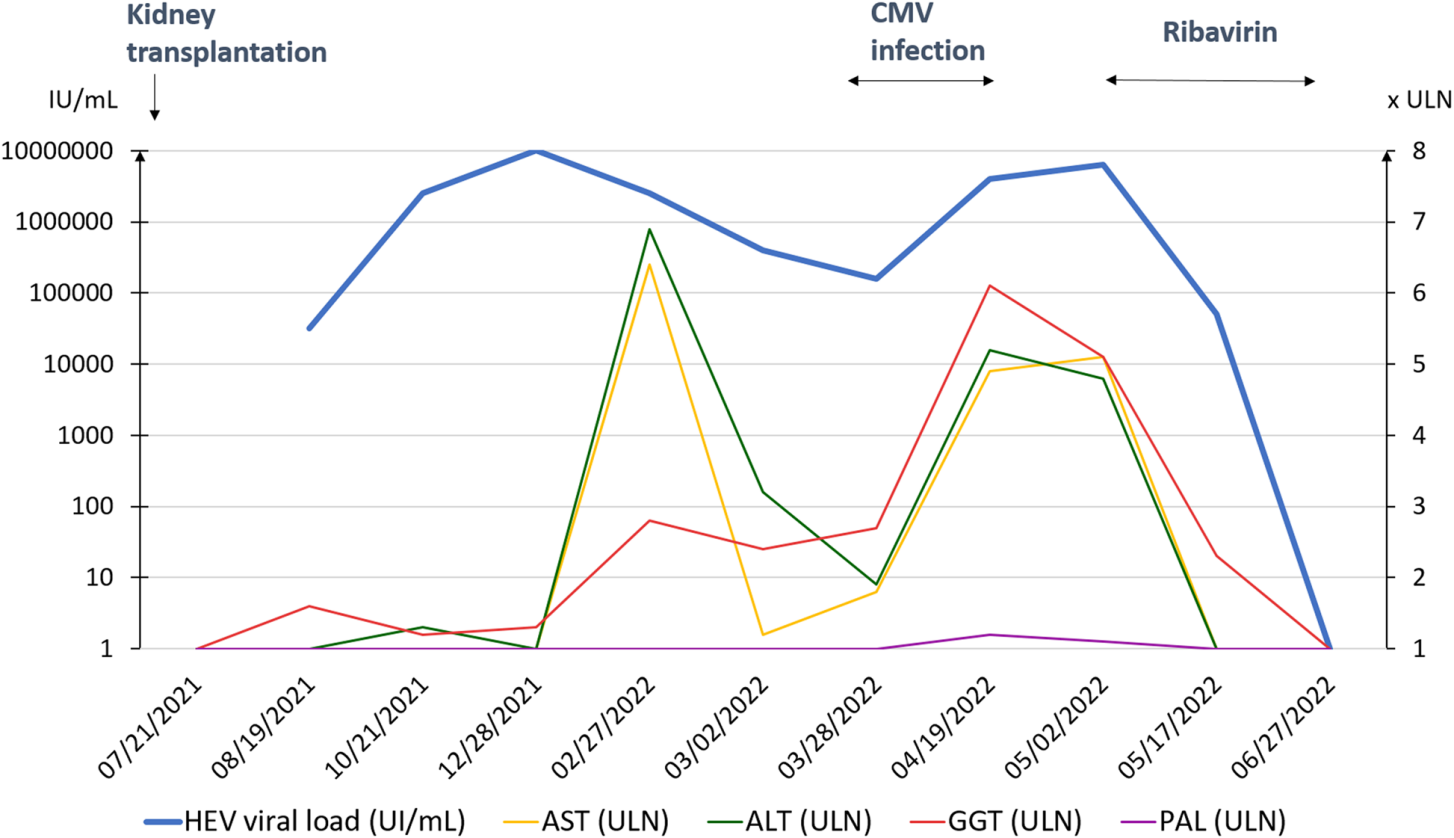
Evolution of HEV viral load (blue curve, Log10IU/mL) of AST (times ULN), of ALT (times ULN), of GGT (times ULN) and ALP (times ULN) in the first year post-kidney transplantation. ULN of laboratory was 35 UI/L for AST and ALT, 40 UI/L for GGT and 105 UI/L for ALP. A result of liver function tests below the ULN has been reported as 1 time ULN in this graph. HEV: hepatitis E virus. AST: aspartate transaminase. ALT: alanine transaminase. CMV: cytomegalovirus. GGT: gamma-glutamyltransferase. ALP: alkaline phosphatase. ULN: upper limit of normal

A woman in her 50s with polycystic kidney and hepatic disease underwent kidney transplantation on 21 July 2021. She received immunosuppressive therapy with anti-thymocyte globulins, tacrolimus, mycophenolate mofetil, and corticosteroids. She presented a negative cytomegalovirus (CMV) serology whereas donor serology was positive. Kidney graft function was stabilized at 98 µmol/L of creatinine 3 months after transplantation, but the patient developed post-transplant diabetes, and two episodes of acute graft pyelonephritis in January and February 2022. On 27 February 2022, she was hospitalized for isolated fever while still being treated with ertapenem for her second episode of acute graft pyelonephritis. Levels of the gamma-glutamyl transferase (GGT), alanine aminotransferase (ALT), and aspartate aminotransferase (AST) were increased to 109, 243, and 225 IU/L, respectively. Alkaline phosphatase (ALP) and bilirubin levels were within reference range. There was no sign of hepatocellular deficiency. The patient had no background of alcohol intake or illicit drug use. The abdominal ultrasonography was normal. HEV RNA detection was positive using a real-time reverse transcription Polymerase Chain Reaction (qPCR) assay, as previously described [23], viral load was 6.5 log_10_IU/mL, and IgM and IgG serologies were negative (Fig. 1).

HEV was determined to be of genotype 3c using the Hepatitis E Virus Genotyping Tool v.1.0 (https://www.rivm.nl/mpf/typingtool/hev/) and by phylogeny (Supplementary Figure S1). No other infectious agents were identified. We then tried to define the mode and time of infection. Questioning did not find evidence for foodborne transmission of HEV during the 10 previous weeks. There was no history of transfusion.

Retrospective examination of biological data revealed that liver function abnormalities dated from August 2021, i.e. during the first month after kidney transplantation. Indeed, we noticed an increase in the GGT level on 10 August 2021. An increase of ALT was subsequently detected on 7 October 2021. Liver function tests were normal before the graft. Additional retrospective analyses performed on available sera detected HEV RNA by qPCR on 19 August 2021 at a titer of 4.5 log_10_IU/mL, while qPCR was negative the day of the transplantation (Fig. 1). In addition, abnormality of liver tests and the presence of HEV RNA in the serum for more than 6 months led us to conclude that it was a case of chronic hepatitis E. Besides, HEV infection occurrence so close to the transplantation raised suspicion of transmission from the donor.

The donor was a man in his 50s admitted to the Intensive Care Unit (ICU) for a cardiac arrest following a severe trauma. He died after Maastricht category III circulatory arrest. The two kidneys and tissues, including bones and cardiac valves, were recovered whereas the liver was not because of suspicion of an ischemic cholangitis. Indeed, liver tests were abnormal on admission to the ICU (ALT 52 IU/L, AST 107 IU/L, GGT 84 IU/L, ALP 61 IU/L). The donor received two packed red blood cells and one pack of platelets before organ procurement.

We retrospectively detected HEV RNA by qPCR in the donor’s serum while HEV IgG and IgM were negative. Viral load was 4.9 log_10_copies/mL. Retrospective qPCR showed that stored health and safety samples of the three blood products received by the organ donor were free of HEV RNA. Based on these findings and their timing, and the 100% identity between HEV sequences obtained from the organ donor and the kidney transplant recipient, in addition to phylogenetic analysis including these sequences (Supplementary Figure S1), we retained the diagnosis of nosocomial HEV transmission through the kidney transplant.

HEV RNA viral load in the transplant recipient was 5.6 log_10_IU/mL on 3 March 2022 and 5.3 log_10_IU/mL on 28 March 2022 (Fig. 1). She was hospitalized on 28 March 2022 for CMV disease with digestive, pulmonary, and hematological involvement. She was treated with ganciclovir from 29 March to 19 April 2022 associated with the decrease of immunosuppressive therapy (reduction of the mycophenolate mofetil dosage by a factor of 4). On 19 April 2022, HEV RNA load had increased to 6.8 log_10_IU/mL. CMV PCR was negative. The patient was thus treated with ribavirin at 800 mg/day from 2 May 2022 for 3 months. On 27 June 2022, liver function tests were normal, and HEV qPCR was negative. Although HEV seroconversion was not observed in August 2022 it was apparent in April 2023 for both IgM and IgG anti-HEV antibodies. HEV RNA tested retrospectively using the Altona RT-PCR assay (Altona Diagnostics, Hamburg, Germany) on a stored stool sample collected as on April 2023 was undetectable. Despite seroconversion, she developed new HEV viremia on June 16 2023, 10 months after ribavirin treatment had ended. Phylogenetic analysis indicated that the strain was the same (Supplementary Figure S1). Her liver function test and fibroscan were normal. Because HEV viremia persisted for 3 months, Ribavirin was reintroduced from October 2023 to January 2024, resulting in HEV viremia clearance. HEV qPCR in the stools was negative in January 2024 (Altona RT-PCR assay).

We conducted an investigation to identify other infected recipients from the same donor. The two kidneys and tissues – as bones and cardiac valves – were transplanted. Mild GGT level increase was observed 9 days after the kidney transplant in the other kidney recipient, but not secondary to HEV infection since blood samples retrospectively tested negative for anti-HEV IgM and HEV RNA just before the transplant as well as 3 and 9 months post-transplant. A left knee medial tibia plateau was grafted 2 months after procurement for the repair of a complex knee fracture in a 25-year-old woman. Anti-HEV IgM and IgG were negative 7.5 months post-osseous graft. In conclusion, HEV transmission occurred in only one of two kidney recipients. Recipients of donor tissues appear to have remained free of infection, subject to missing data.

## Discussion

Here we describe a case of HEV-3 infection transmitted by a kidney transplant donor. This case is distinguished by the mode of HEV transmission that is classically zoonotic in Europe, mostly foodborne, with pigs and wild boars as the main reservoir.

While HEV transplant transmission had not been documented until 2012 [2], several cases have been described since then. The first case of liver transplant transmission was described in 2012 in Germany [27]. Liver cirrhosis developed within 15 months and the patient died of septic shock. A second case of liver transplant transmission was described in 2018 in China which was part of an outbreak that affected five organ transplant recipients who shared the same donor and were all diagnosed at the stage of chronic hepatitis E [29]. The liver transplant recipient developed severe hepatitis with progressive portal hypertension. Regarding HEV kidney transplant transmission, four cases had been reported: two in France in 2017 and two in China in 2018 [28, 29]. In these four cases, HEV sequences from the transplant recipients were identical with each other and with that of the common donor, indicating donor-derived infection [27, 28]. HEV was of genotype 3 for the European cases and of genotype 4 for the Chinese cases. A case of HEV infection presumed to have been acquired through lung transplantation, was described in the USA in 2017 [30]. Supporting evidence included the positive anti-HEV IgG and IgM testing of the donor’s serum and the patient’s persistent transaminitis that began after transplantation, even though the donor was negative for HEV RNA. HEV infection was diagnosed in the lung transplant recipient at the cirrhosis stage, simultaneously to HEV meningoencephalitis, 11 years post-transplantation. Finally, a recent publication by Ushiro-Lumb et al*.*, has further documented transmission by organ donation diagnosed through the UK's systematic donor screening policy [31]. Between 2017 and 2022, 9500 deceased potential organ donors were screened with nine confirmed viraemic cases identified (incidence of 0.94 per 1000). Among the 20 organ recipients from the 8 proceeding donors, 14 cases of infection were diagnosed: in 5 liver transplant recipients and 9 kidney transplant recipients. As evidence of HEV organ donor transmission accumulates, it is possible that organ donor-derived HEV infections are underestimated in endemic areas, since acute and chronic hepatitis E in immunocompromised patients remain underdiagnosed [19, 21].

Secondary HEV transmission between humans is rare [5] and the scarcity of this event might be notably explained by dose-dependence and a high infectious dose [32, 33]. The organ donor-derived HEV infections raise the question of infectious dose. As liver is the target and the main reservoir of HEV during hepatitis, HEV transmission by liver transplantation is not surprising. In the German study by Schlosser et al*.,* antibody screening and RT-PCR were negative in the donor but HEV RNA was reported to be “in high concentration” in the liver [27]. In the Chinese study by Sridhar and colleagues, HEV was also detected in the donor liver and viral load was 288 copies/reaction [29]. In the English series by Ushiro-Lumb et al*.,* all liver transplant recipients from a donor with HEV viremia, even very low at 100 IU/mL, had an infection (except for one recipient who presented a transient viremia in the context of liver graft withdrawal on day 5) [31]. In the cases of HEV transmission by kidney, lung, and heart transplants, it can be hypothesized that HEV was transmitted by the blood remaining in the different organs after washout. The risk of transfusion-transmitted HEV infection has been found to be closely related to the plasma volume of blood components [34, 35]. In a study performed in southeast England, Tedder et al*.* reported that the HEV-infected blood donations associated with transmission exhibited a median viral titer of 4.53 log_10_IU/ml and a lowest titer leading to infection of 2.61 log_10_IU/ml [36]. Significant viremia was retrospectively detected on archived serum from each organ donor collected on the organ retrieval day and exceeded the minimum infectious titer seen in blood products, being 6.5 log_10_IU/mL for the French kidney donor, 5.3 log_10_IU/mL for the Chinese organ donor, and 4.9 log_10_IU/mL for our donor. Among the fourteen patients receiving kidney transplant from HEV positive English donors, five developed no infection, and their donors had a viral load of less than 500 IU/mL. Nevertheless, five developed HEV infections and their donor's viral load ranged significantly, from 111 IU/mL to 4.9 log IU/mL, suggesting the importance of transmission factors other than viral load [31]. Infectious HEV circulates in peripheral blood as enveloped particles that appear to be the major type of HEV in blood and therefore the primary cause of blood-transmitted HEV infections, while non-enveloped particles excreted in stools are the most infectious [37]. In the case presented here, tested recipients of tissues remained free of infection. In addition, a recent literature review found no case of HEV infection transmitted through cell and tissue allografts [38].

Considering the clinical presentation of the nine cases of organ donor-derived HEV infections without systematic screening published in the literature and found in our case, HEV infection was diagnosed at the chronic hepatitis stage in all patients, at post-mortem for two ( [27, 29], present case) and after a mean of 211 days after transplantation for seven ( [28, 29], present case). HEV RNA positivity determined retrospectively has been reported after a mean time of 57 days and as soon as 21 days after transplantation on available sera for four patients ( [27, 29], present case). Two cases developed liver cirrhosis [27, 30], and one presented severe hepatitis with progressive portal hypertension [29]. Although the rate of progression to chronicity varied between the different studies, HEV infections overall became chronic in about two-thirds of SOTRs [21, 22, 24], while almost all the cases of organ donor-transmitted HEV infection in SOTRs are described as having become chronic. Furthermore, we observed a relapse of chronic hepatitis despite ribavirin treatment and sero-conversion in our case.

Organ donor-transmitted HEV infection in SOTRs is characterized by an infection on the day of transplantation, probably at the worst juncture for achieving an immune response against HEV. In fact, in previous studies in SOTRs, there was no trend for an association between initial viral load and chronic HEV infection [19, 21]. Such an observation, however, largely depends on the clinical samples available for testing. It is possible that the temporality between HEV infection and organ transplantation and the introduction of immunosuppressive treatment in organ donor-transmitted HEV infection challenges this result. There have been studies on the implications of immunosuppressive drugs (apart from anti-T polyclonal antibodies) for treatment in chronic hepatitis E [39, 40]. Immunosuppressive induction treatment led as expected to the quasi-complete depletion of lymphocytes in our patient (total lymphocyte count of 0.97 G/L before and 0.06 G/L several hours after transplant). This highlights an inadequate defense against HEV provided by innate immunity. It should be noted that clinical presentation as mild hepatitis then moderate chronic hepatitis looks like the HEV infection of athymic nude rats, resulting in mild alterations of liver enzyme levels, lack of hepatic injury, and persistent hepatitis, as reported by Debing et al. [41].

All the cases of organ donor-transmitted HEV infection have been diagnosed at the chronic hepatitis stage. If this evolution was undoubtedly favored by immunosuppression, the lack of attention to mild or moderate liver function test impairment at the beginning of the hepatitis course should also be highlighted, because it is typical of E hepatitis in SOTRs compared to immunocompetent patients, and has been associated with chronic HEV infection development [19, 21, 42]. Two French SOTR cohorts estimated that 6 to 8% of patients with biological liver abnormalities had hepatitis E, which reflects the high HEV endemicity level in France [16, 43]. In view of the potential severity of liver damage, any biological liver abnormalities in SOTRs should be investigated for HEV by qPCR, the diagnostic method of preference for on-going HEV infection [19, 21, 43]. Furthermore, in the absence of any abnormal liver function tests, we recommend systematic annual screening for hepatitis E in SOTRs, because we showed that HEV monitoring in KTRs is clinically relevant especially in high endemic regions [44]. Moreover, this has been estimated to be cost-effective in a UK study, expecting to avoiding 7 cases of cirrhosis and 3.5 cases of cirrhosis-related death per 1000 screened patients [45]. When considering the time to diagnosis, any diagnosis of HEV infection in SOTRs within the first year of transplantation should raise the possibility of transmission from the organ donor.

The hope for a better prevention of HEV infection in immunocompromised patients may notably depend on the availability of safe and effective vaccines. The only approved vaccine (Hecolin) was licensed in China in 2011 [5]. Because neutralizing anti-HEV antibodies produced in response to a natural infection mainly target the capsid protein, it has been suggested that neither pre-exposure to HEV nor ORF2-based HEV vaccines such as Hecolin confer protection against enveloped HEV virions [37]. However, vaccination with Hecolin in rabbits prior to administration of immunosuppressants fully protected them against HEV genotype 3 and 4 infections, whereas only partial protection was achieved when the animals were already receiving immunosuppressants [46]. These data could argue in favor of preferentially vaccinating patients on the waiting list for organ transplantation [39]. As HEV vaccines are not commercialized worldwide, as evidence of HEV organ donor transmission accumulates in endemic areas, and as mild liver function test impairment potentially related to hepatitis E in immunocompromised are insufficiently considered, we think that systematic screening of organ donors – at least in HEV hyperendemic areas such as ours [47] – should be implemented independently of liver function abnormalities. This would be in line with screening already performed in the UK and Spain for deceased and living donors, regardless of HEV incidence [44, 48, 49]. Recommendations from The British Transplantation Society and the UK Advisory Committee for the Safety of Blood, Tissues and Organs (SaBTO), in place since 2017, state that all organ donors must be screened for hepatitis E viremia using HEV-Nucleic Acid Amplification Testing.

In France, a similar policy has been implemented no later than January 2024. The timing of HEV systematic screening depends on the type of donor. In the case of a living kidney donor, screening will be carried out the week before the kidney donation according to new French recommendation. If it is positive, the transplant will be cancelled. In Marseille, we also screen HEV RNA during the initial assessment of donors. In the case of a deceased donor, screening will be carried out emergency prior to the kidney donation, with the constraint that the screening result may not be available at the time of donation: the donor's HEV status will be determined by qPCR within 48 h. When assessing the risk–benefit balance for transplantation, the donor's liver function must be carefully evaluated. In this context, the detection of viremia is unlikely to be an absolute contra-indication to use of an organ from a donor but will certainly inform clinical management decisions post-transplant.

## Conclusion

Documented cases of HEV infection derived from organ donation should not be overlooked. Given the potential severity of HEV infection in immunocompromised patients, the initial presentation as moderate hepatitis, and the potential delay in diagnosis, we believe that systematic screening of organ donors is valuable for both deceased and living donors.

### Supplementary Information

Additional file 1: Supplementary Figure S1. Phylogeny reconstruction based on a fragment of the ORF2 gene.

## Data Availability

No datasets were generated or analysed during the current study.

## Abbreviations

ALP: Alkaline phosphatase
ALT: Alanine transaminase
AST: Aspartate transaminase
CMV: Cytomegalovirus
GGT: Gamma-glutamyltransferase
HEV: Hepatitis E virus
ICU: Intensive Care Unit
qPCR: Real-time Polymerase Chain Reaction
SOTRs: Solid organ transplant recipients
ULN: Upper limit of normal

## References

[1] Dalton HR, Bendall R, Ijaz S, Banks M (2008). Hepatitis E: an emerging infection in developed countries. Lancet Infect Dis.

[2] Kamar N (2012). Hepatitis E. Lancet.

[3] Mansuy JM (2015). Seroprevalence in blood donors reveals widespread, multi-source exposure to hepatitis E virus, southern France, October 2011. Euro Surveill.

[4] Mansuy JM (2016). A nationwide survey of hepatitis E viral infection in French blood donors. Hepatology.

[5] Kamar N (2017). Hepatitis E virus infection. Nat Rev Dis Primers.

[6] Nagashima S (2017). Characterization of the Quasi-Enveloped Hepatitis E Virus Particles Released by the Cellular Exosomal Pathway. J Virol.

[7] Purdy MA, et al. ICTV Virus Taxonomy Profile: Hepeviridae 2022. J Gen Virol. 2022;103(9). 10.1099/jgv.0.001778 PMC12642825 36170152

[8] Rivero-Juarez A (2022). Orthohepevirus C infection as an emerging cause of acute hepatitis in Spain: First report in Europe. J Hepatol.

[9] Sridhar S (2022). Hepatitis E Virus Species C Infection in Humans. Hong Kong Clin Infect Dis.

[10] Lee G-H (2016). Chronic infection with camelid Hepatitis E virus in a liver transplant recipient who regularly consumes camel meat and milk. Gastroenterology.

[11] Reuter G, Boros Á, Pankovics P (2020). Review of Hepatitis E virus in rats: evident risk of species Orthohepevirus C to Human Zoonotic Infection and Disease. Viruses.

[12] Rein DB, Stevens GA, Theaker J, Wittenborn JS, Wiersma ST (2012). The global burden of hepatitis E virus genotypes 1 and 2 in 2005. Hepatology.

[13] Nimgaonkar I, Ding Q, Schwartz RE, Ploss A (2018). Hepatitis E virus: advances and challenges. Nat Rev Gastroenterol Hepatol.

[14] Pérez-Gracia MT, Suay-García B, Mateos-Lindemann ML (2017). Hepatitis E and pregnancy: current state. Rev Med Virol.

[15] Kumar S, Subhadra S, Singh B, Panda BK (2013). Hepatitis E virus: the current scenario. Int J Infect Dis.

[16] Kamar N (2008). Hepatitis E virus and chronic hepatitis in organ-transplant recipients. N Engl J Med.

[17] Gérolami R, Moal V, Colson P (2008). Chronic hepatitis E with cirrhosis in a kidney-transplant recipient. N Engl J Med.

[18] Fujiwara S (2014). Chronic hepatitis E: a review of the literature. J Viral Hepat.

[19] Legrand-Abravanel F (2010). Characteristics of autochthonous hepatitis E virus infection in solid-organ transplant recipients in France. J Infect Dis.

[20] Sridhar S (2018). Rat Hepatitis E Virus as Cause of Persistent Hepatitis after Liver Transplant. Emerg Infect Dis.

[21] Kamar N (2011). Factors associated with chronic hepatitis in patients with hepatitis E virus infection who have received solid organ transplants. Gastroenterology.

[22] Buescher G (2021). Hepatitis E seroprevalence and viremia rate in immunocompromised patients: a systematic review and meta-analysis. Liver Int.

[23] Moal V (2013). Infection with hepatitis E virus in kidney transplant recipients in southeastern France. J Med Virol.

[24] uropean Association for the Study of the Liver. Electronic address: easloffice@easloffice.eu & European Association for the Study of the Liver (2018). EASL Clinical Practice Guidelines on hepatitis E virus infection. J Hepatol.

[25] Gallian P (2019). Transfusion-Transmitted Hepatitis E Virus Infection in France. Transfus Med Rev.

[26] Boland F, Martinez A, Pomeroy L, O’Flaherty N (2019). Blood Donor Screening for Hepatitis E Virus in the European Union. Transfus Med Hemother.

[27] Schlosser B (2012). Liver transplant from a donor with occult HEV infection induced chronic hepatitis and cirrhosis in the recipient. J Hepatol.

[28] Pourbaix A, et al. Evidence of hepatitis E virus transmission by renal graft. Transpl Infect Dis. 2017;19(1). 10.1111/tid.12624 27775205

[29] Sridhar S (2019). Donor-Derived Genotype 4 Hepatitis E Virus Infection, Hong Kong, China, 2018. Emerg Infect Dis.

[30] Murkey JA (2017). Hepatitis E Virus-Associated Meningoencephalitis in a Lung Transplant Recipient Diagnosed by Clinical Metagenomic Sequencing. Open Forum Infect Dis.

[31] Ushiro-Lumb I (2023). Impact of Hepatitis E Virus Screening in the UK Deceased Organ Donor Population. Transpl Int.

[32] Purcell RH, Emerson SU (2008). Hepatitis E: an emerging awareness of an old disease. J Hepatol.

[33] Dreier J, Knabbe C, Vollmer T (2018). Transfusion-Transmitted Hepatitis E: NAT Screening of Blood Donations and Infectious Dose. Front Med (Lausanne).

[34] Laperche S (2023). Seven years (2015–2021) of blood donor screening for HEV-RNA in France: lessons and perspectives. Blood Transfus.

[35] Hewitt PE (2014). Hepatitis E virus in blood components: a prevalence and transmission study in southeast England. Lancet.

[36] Tedder RS (2017). Hepatitis E risks: pigs or blood-that is the question. Transfusion.

[37] Costafreda MI, Sauleda S, Rico A, Piron M, Bes M (2022). Detection of Nonenveloped Hepatitis E Virus in Plasma of Infected Blood Donors. J Infect Dis.

[38] Villalba R, Mirabet V (2022). Risk assessment of hepatitis E transmission through tissue allografts. World J Gastrointest Pathophysiol.

[39] Ma Z, de Man RA, Kamar N, Pan Q (2022). Chronic hepatitis E: Advancing research and patient care. J Hepatol.

[40] Wang Y, Metselaar HJ, Peppelenbosch MP, Pan Q (2014). Chronic hepatitis E in solid-organ transplantation: the key implications of immunosuppressants. Curr Opin Infect Dis.

[41] Debing Y (2016). A rat model for hepatitis E virus. Dis Model Mech.

[42] Gérolami R, Moal V, Picard C, Colson P (2009). Hepatitis E virus as an emerging cause of chronic liver disease in organ transplant recipients. J Hepatol.

[43] Moal V (2013). Hepatitis E virus serological testing in kidney transplant recipients with elevated liver enzymes in 2007–2011 in southeastern France. Diagn Microbiol Infect Dis.

[44] Guidelines from the expert advisory committee on the Safety of Blood, Tissues and Organs (SaBTO) on measures to protect patients from acquiring hepatitis E virus via transfusion or transplantation. https://assets.publishing.service.gov.uk/government/uploads/system/uploads/attachment_data/file/680297/Hepatitis_E_Guidelines.pdf.

[45] Ankcorn MJ, Tedder RS, Cairns J, Sandmann FG (2020). Cost-Effectiveness Analysis of Screening for Persistent Hepatitis E Virus Infection in Solid Organ Transplant Patients in the United Kingdom: a model-based economic evaluation. Value Health.

[46] He Q (2022). Immunocompromised rabbit model of chronic HEV reveals liver fibrosis and distinct efficacy of different vaccination strategies. Hepatology.

[47] Moal V (2015). Systematic serological testing for hepatitis E virus in kidney transplant recipients. J Clin Microbiol.

[48] Guidelines for Hepatitis E & Solid Organ Transplantation. https://bts.org.uk/wp-content/uploads/2017/06/BTS_HEV_Guideline-FINAL.pdf.

[49] Rivero-Juárez A (2020). Executive summary: Consensus document of the diagnosis, management and prevention of infection with the hepatitis E virus: Study Group for Viral Hepatitis (GEHEP) of the Spanish Society of Infectious Diseases and Clinical Microbiology (SEIMC). Enferm Infecc Microbiol Clin (English ed).

