# Supplementary material for: The question of screening organ donors for hepatitis e virus: a case report of transmission by kidney transplantation in France and a review of the literature
Source: Virol J. 2024 Jun 12;21:136. doi: 10.1186/s12985-024-02401-2 (PMC11167830; doi:10.1186/s12985-024-02401-2)

**Supplementary Material**

**Supplementary Figure S1. Phylogeny reconstruction based on a fragment of the ORF2 gene**

The fragment of the ORF2 gene encoding for the capsid protein was 315-nucleotides long and corresponded to nucleotides 6,015-6,329 of the HEV genome GenBank accession no. FJ705359. The HEV sequences obtained by Sanger sequencing from the present cases are indicated by a white bold font, a black background, and labeled “Donor” or “Recipient”. The ten sequences with the highest BLAST scores recovered from the NBCI GenBank nucleotide sequence databases (http://www.ncbi.nlm.nih.gov/nucleotide/), indicated by bold font and “10BBH” (for 10 best BLAST hit), were incorporated in the phylogeny reconstruction, in addition to reference sequences for HEV genotypes [1]. Nucleotide alignments were performed using the MUSCLE software (http://www.ebi.ac.uk/Tools/msa/muscle/). The evolutionary history was inferred in the MEGAX software (http://www.megasoftware.net/) using the Neighbor-Joining and the Kimura 2-parameter methods. The percentage of replicate trees in which the associated taxa clustered together in the bootstrap test (1,000 replicates) is shown next to the branches. The tree is drawn to scale, with branch lengths in the same units as those of the evolutionary distances used to infer the phylogenetic tree; the scale bars indicate the number of nucleotide substitutions per site. Bootstrap values >50% are labeled on the tree.

**References**

1. D.B. Smith, J. Izopet, F. Nicot, et al. Update: proposed reference sequences for subtypes of hepatitis E virus (species Orthohepevirus A). J Gen Virol 2020;101:692-8. doi 10.1099/jgv.0.001778


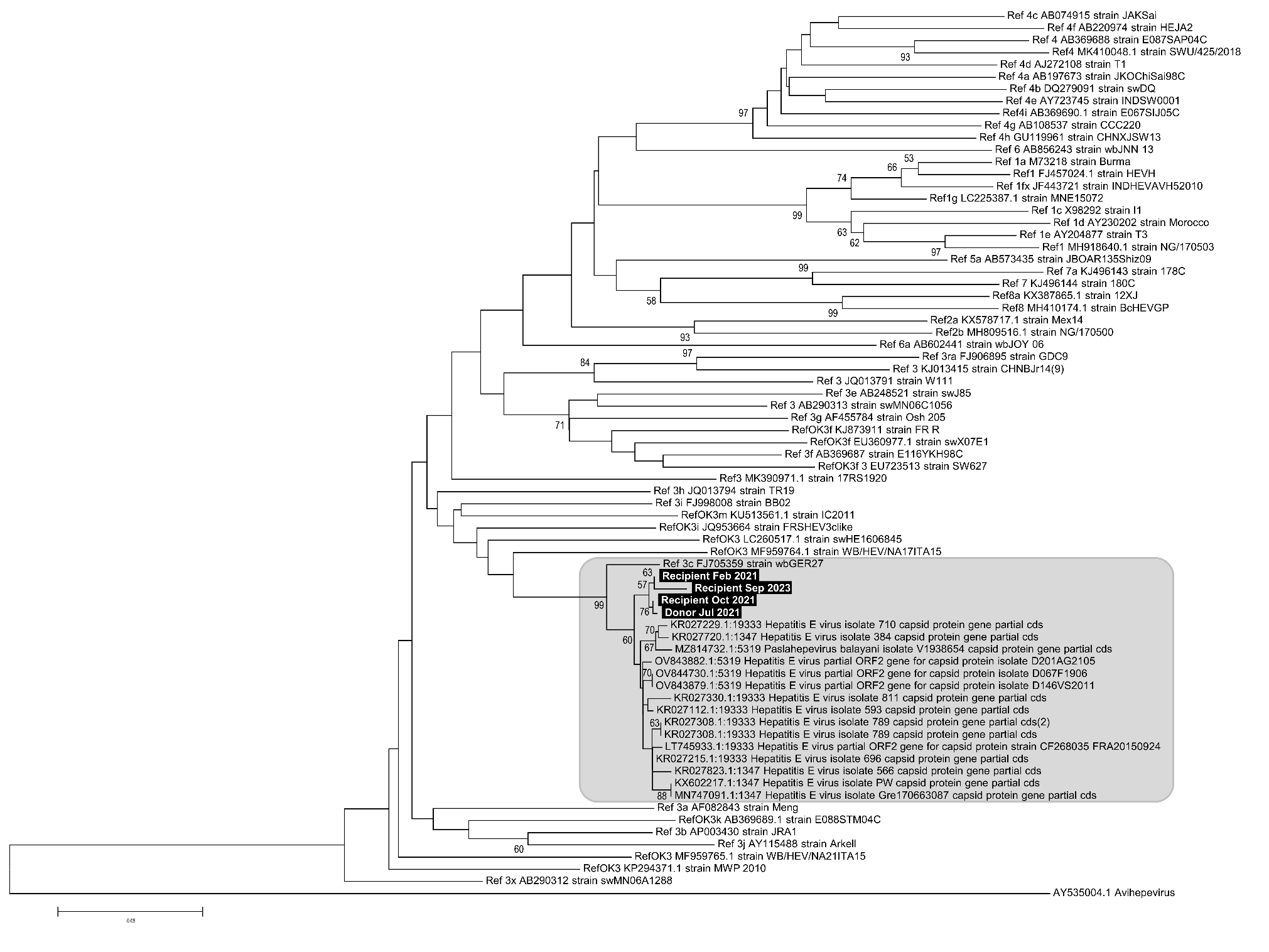

Supplement: Supplementary file 1 — Additional file 1: Supplementary Figure S1. Phylogeny reconstruction based on a fragment of the ORF2 gene. [file 12985_2024_2401_MOESM1_ESM.docx]
